# Human ALKBH6 Is Required for Maintenance of Genomic Stability and Promoting Cell Survival During Exposure of Alkylating Agents in Pancreatic Cancer

**DOI:** 10.3389/fgene.2021.635808

**Published:** 2021-04-07

**Authors:** Shengyuan Zhao, Rodan Devega, Aaliyah Francois, Dawit Kidane

**Affiliations:** Division of Pharmacology and Toxicology, College of Pharmacy, Dell Pediatric Research Institute, The University of Texas at Austin, Austin, TX, United States

**Keywords:** alkbh6, alkylating DNA damage, pancreatic cancer, DNA repair, Alkb *E. coli*

## Abstract

Alpha-ketoglutarate-dependent dioxygenase (ALKBH) is a DNA repair gene involved in the repair of alkylating DNA damage. There are nine types of ALKBH (ALKBH1-8 and FTO) identified in humans. In particular, certain types of ALKBH enzymes are dioxygenases that directly reverse DNA methylation damage via transfer of a methyl group from the DNA adduct onto α-ketoglutarate and release of metabolic products including succinate and formaldehyde. Here, we tested whether ALKBH6 plays a significant role in preventing alkylating DNA damage and decreasing genomic instability in pancreatic cancer cells. Using an *E. coli* strain deficient with ALKB, we found that ALKBH6 complements ALKB deficiency and increases resistance after alkylating agent treatment. In particular, the loss of ALKBH6 in human pancreatic cancer cells increases alkylating agent-induced DNA damage and significantly decreases cell survival. Furthermore, *in silico* analysis from The Cancer Genome Atlas (TCGA) database suggests that overexpression of ALKBH6 provides better survival outcomes in patients with pancreatic cancer. Overall, our data suggest that ALKBH6 is required to maintain the integrity of the genome and promote cell survival of pancreatic cancer cells.

## Introduction

Pancreatic cancer is the seventh leading cause of cancer-related death in the world (Bray et al., 2020). The global cancer statistics estimate 495,773 new cases and 466,003 deaths in 2020. Pancreatic cancer patients are often diagnosed at late stages of the disease, and approximately 20–30% of patients experience relapse after surgical therapy (Mahipal et al., 2015). The 5-year overall survival of patients is less than 10% (Conroy et al., 2011; Von Hoff et al., 2013). The extremely low 5-year overall survival of patients is, in part, due to pancreatic cancer cells having several mechanisms of resistance to different chemotherapeutic treatments, one of which is their capacity to efficiently repair alkylating agent-induced DNA damage. Importantly, alkylating agents induce DNA damage at different genomic sites, which subsequently leads to lethal and/or mutagenic DNA base lesions. The major repair mechanisms for alkylation damage are direct DNA repair, base excision repair (BER), and mismatch repair (MMR) (Kondo et al., 2010). It is critical to uncover the molecular mechanisms that contribute to carcinogenesis and to identify an effective novel target for therapies directed toward pancreatic cancer.

Direct DNA repair comprises several types of DNA repair enzymes that reversibly remove alkyl groups from damaged DNA bases through oxidative dealkylation processes. The α-ketoglutarate- and Fe (II)-dependent dioxygenases remove alkyl groups from DNA and proteins via oxidative demethylation (Mishina et al., 2004). In several studies in *Escherichia coli*, the induction of the Ada response with alkylating agents results in increased expression of different genes including *ADA*, ALKA, ALKB, and AIDB (Lindahl et al., 1988). In particular *Escherichia coli* (*E. coli*) have shown that certain types of alkylating agent-induced DNA base damage is repaired by AlkB dioxygenase via direct reversal repair pathways (Sedgwick et al., 2007; Yi and He, 2013). Together with *O*^6^-methylguanine-DNA methyltransferase (MGMT), the ALKB dioxygenase removes alkyl lesions to protect the genome from mutagenic and/or cytotoxic consequences (Zhang et al., 1993; Rose et al., 2011). Several alkylating agents are commonly used in chemotherapy but may induce DNA damage to cancer cells as well as normal cells. Based on alkylating mechanisms, these agents can be divided as SN1 and SN2. *N*-methyl-*N*’-nitro-*N*-nitrosoguanidine (MNNG), a common SN1 agent, predominantly induces *O*^6^-methylguanine (O^6^mG) and *N*^3^-methyladenine (m^3^A) lesions, which then block the DNA replication fork and generate genotoxic double-stranded DNA (DSBs) breaks (Loechler et al., 1984; Shah and Gold, 2003). In comparison, the SN2 agent methyl methane sulfonate (MMS) induces *N*^1^-methyladenine (m^1^A) and *N*^3^-methylcytosine (m^3^C) lesions, preferably in single-stranded DNA (ssDNA). Overall, accumulation of these unrepaired DNA base lesions and their repair intermediates can lead to cell death (van den Born et al., 2009; Dietlein et al., 2014).

In *E. coli*, the ALKB (EcALKB) protein is involved in DNA and RNA repair in response to environmental stress (Nieminuszczy and Grzesiuk, 2007). Several biochemical and genetic studies have demonstrated that the EcALKB protein initiates repair by promoting oxidative demethylation of the alkyl DNA base lesions, including m^1^A and m^3^C lesions (Falnes et al., 2002; Trewick et al., 2002). In contrast, in the human genome, there are nine genes that encode EcALKB homologs, including ALKBH1-8 and FTO (fat mass and obesity-associated protein) (Aravind and Koonin, 2001; Sedgwick et al., 2007; Ougland et al., 2015). So far, a significant number of human ALKBH proteins (ALKBH1, ALKBH2, ALKBH3, and FTO) have been characterized *in vitro* to show DNA repair activity and epigenetic as well as RNA post transcription modification on methylated DNA/RNA substrates (Duncan et al., 2002; Aas et al., 2003; Westbye et al., 2008). In particular, ALKBH2 (Nay et al., 2012) and ALKBH3 (Sundheim et al., 2006) complement ALKB-deficient *E. coli in vivo* (Aas et al., 2003). In addition, ALKBH2 and ALKBH3 are involved in the repair of alkylating DNA damage in both cell culture and animal models to protect the integrity of the genome (Calvo et al., 2012; Fu and Samson, 2012; Aihara et al., 2016). Studies conducted in a transgenic knockout mouse model show that ALKBH2 repairs m^1^A lesions (Ringvoll et al., 2006), while ALKBH3 plays a role in the repair of m^3^C lesions in human cells (Dango et al., 2011). ALKBH2 and ALKBH3 use the same α-KG/Fe(II)-dependent mechanism to oxidize alkyl groups on DNA base lesions, subsequently removing the damage and restoring normal DNA bases (Zheng et al., 2014; Fedeles et al., 2015; Aihara et al., 2016). However, some of the other α-KG/Fe(II)-dependent ALKBH proteins are involved in various other biochemical pathways including RNA metabolism, histone demethylation, and fatty acid metabolism (Rajecka et al., 2019). For example, ALKBH8 and ALKBH5 are involved in processing tRNAs and modifying N(6)-methyladenosine (N^6^-mA) in mRNA, respectively (Fu et al., 2010). In contrast, ALKBH1 is an important nuclear eraser of *N*^6^-mA in unpairing regions of DNA of the mammalian genome (Zhang et al., 2020). These findings suggest additional roles of mammalian ALKBH enzymes in RNA post transcript modification and regulations of epigenetic markers.

However, the role of ALKBH6 in the maintenance of genomic stability and protection from alkylating DNA damage is unknown. In this study, we show that human ALKBH6 is required to prevent genomic instability and to prevent the MMS-induced cytotoxicity seen in pancreatic cancer. Our data show that siRNA silencing of ALKBH6 in pancreatic cancer cell lines increases DSBs and sensitivity to MMS but not MNU. Moreover, our TCGA data analysis shows that a majority of tumor tissues significantly overexpress ALKBH6, as compared to normal tissue, and show poor overall survival in the selected types of human cancers, thus suggesting a pro-carcinogenic role of these proteins (Tan et al., 2015). Specifically, an approximate 4% genetic alteration and 28% mRNA overexpression of ALKBH6 is detected in pancreatic cancer cells compared to other ALKBH genes. In this study, overexpression of ALKBH6 genes improves pancreatic cancer survival rates, compared to pancreatic cancer patients with low expression. However, ALKBH6 overexpression in p53 mutant tumors is associated with poor overall survival. Altogether, these results suggest that ALKBH6 is involved in maintaining genomic integrity during SN2 alkylating agent-mediated DNA damage, and also provides a more favorable prognosis in the overall survival of pancreatic cancer patients.

## Materials and Methods

### Bacterial Strains and Media

The following *E. coli* K12 strains (a gift from Dr. Elżbieta Grzesiuk) were used: AB1157 [argE3, hisG4, leuB6, Δ(gpt -proA)62, thr -1, ara -1, galK2, lacY1, mtl-1, xylA5, thi-1, rpsL31, glnV44, tsx-33, rfbD1, mgl-51, kdgK51] as wild-type (WT: alkB*^+^*); BS87 (as AB1157 but alkB117:Tn3) as *alkB*^–^ (Sedgwick, 1992).

### Construction of Plasmids for ALKBH6 Expression

A human cDNA pool was constructed by reverse transcription-PCR (RT-PCR) with the Transcriptor High Fidelity cDNA Synthesis Kit (Cat. 5081955001, Sigma Aldrich, St. Louis, MO) from RNA extracted from immortalized non-transformed gastric epithelial cell (GES-1). A 650-bp cDNA, encoding the human ALKBH6 open reading frame (nt 280–786 of XM_024451747), was amplified using PCR (Platinum Taq, Invitrogen, Carlsbad, CA). The primers are: 5′-GCGAAGCTTTCACTTGCCCAGCAGG-3′, and 5′-GCTCTAGAGCGGAAATGGCTGGGAG-3′. The amplified product was cloned into pBAD24 (Addgene) as an *Xba*I-*Not*I fragment. The recombinant plasmid was transformed into BS87 with electroporation to construct BS87-pBAD24-ALKBH6, and the expression of ALKBH6 was induced by the addition of 0.1% arabinose (Cat. A3256, Sigma Aldrich).

### Quantitative Growth Curve and Colony-Forming Assay of *E. coli*

WT, ALKB^–^, and transformed *E. coli* were grown in 3 ml of LB broth (Cat. L3022, Sigma Aldrich) at 37°C overnight, shaking at 250 rpm. For growth curve analysis, 10 μl of bacteria culture was added to 5 ml of LB broth containing 1 mM of MMS or 1 mM of MNU with 0.1% arabinose and was allowed to grow at 37°C overnight, again shaking at 250 rpm. The optical density at 600 nm (OD600) was determined every 30 min until the observed growth curve reached a plateau state. For the colony-forming assay, the bacteria culture following overnight growth was diluted to 10^3^ cells/ml using OD600 quantification. Next, 100 μl of the diluted culture was spread on LB plates containing MMS (0–3 mM) or MNU (0–3 mM) with 0.1% arabinose and were allowed to grow overnight at 37°C overnight. The colonies were counted the next day.

### Small Interfering RNA (siRNA) for Silencing of ALKBH6

ON-TARGETplus human ALKBH6 siRNA duplexes (Cat. L-015030-01-0005) were purchased from Dharmacon Research Inc. (Lafayette, CO). All siRNAs were suspended in RNase-free water to achieve a concentration of 5 μM for each siRNA. A final concentration of 25 nM for siRNA was used for subsequent experiments. The siRNA sequences are as follows: J-015030-09: 5′-GGACGCUGGUGGACGGAUU; J-015030-10: 5′-GCUGUGACUCCGCGACCUA; J-015030-11: 5′-AGGAGUAUUUGCUUCGACA; J-015030-12: 5′-GCAAGGA GUUGGUGUUGAU.

### Cell Lines and Drug Treatment

Human pancreatic cancer cell lines MIA-PaCa-2 and BXPC3 (a gift from Dr. John DiGiovanni) were maintained in EMEM media supplemented with 10% Fetal Bovine Serum (FBS), 1% penicillin/streptomycin, and 1% L-glutamine at 37°C in a 5% CO_2_ environment. Methyl methanesulfonate (MMS, Cat. 129925, Sigma-Aldrich, St. Louis, MO) and *N*-nitroso-*N*-methylurea (MNU, Cat. N2939, Spectrum Chemical, New Brunswick, NJ) were dissolved in DMSO, and stored at −20°C before use. Forty-eight hours after siRNA treatment, 70% of the confluent cells were treated with either MMS or MNU at various concentrations for 24 h in culture. The cells were then washed with PBS and used for further analysis.

### Clonogenic Survival Assay

A clonogenic survival assay was performed according to the previously published protocol, including minor modifications (Franken et al., 2006). Both the control and ALKBH6-siRNA silenced cells were plated in six-well plates at a density of 500 cells per well and cultured to allow adherence overnight. Seventy percent of confluent cells were then treated with MMS (0–5 mM), or MNU (0–5 mM) for 24 h. Following treatment, the cells were then washed with PBS and supplemented with fresh growth media and allowed to grow for an additional 10 days. The cells were then stained with 0.25% crystal violet in an 80% methanol solution for 30 min. The colonies were finally counted and scored using visual techniques.

### Western Blotting

Cells were lysed with radioimmunoprecipitation assay (RIPA) buffer supplemented with a protease inhibitor (Cat. 25765800, Sigma Aldrich) and a phosphatase inhibitor (Cat. P5726, Sigma Aldrich). Protein concentration was measured using a BCA kit as described by the manufacturer (Cat# 23250 Thermo Fisher Scientific, United States). After denaturing the samples at 95°C for 5 min, 30 μg of the protein samples were separated using SDS-PAGE and transferred onto nitrocellulose membranes (Cat. 1620112, Bio-Rad, Hercules, CA). Next, the membranes were blocked with 5% BSA for 1 h, and then incubated with primary antibodies against γH_2_AX (Cat. 07-164, Millipore, Burlington, MA), ALKBH6 (Cat. ab170186, Abcam, Cambridge, MA; antibody dilution 1:1,000), and α-tubulin (Cat. 2125S, Cell Signaling, Danvers, MA; antibody dilution 1:500) overnight. The following day, the membranes were washed with PBST and incubated with anti-mouse (Cat. NXA931, GE healthcare, Chicago, IL) or anti-rabbit (Cat. NA934V, GE Healthcare) secondary antibody for 2 h before developing with ECL substrates (Bio-rad, 170506). The gel images were captured using A Chem-DocXRS image acquisition machine (Bio-Rad).

### Immunofluorescence

The 5 × 10^4^ cells were seeded in cover slips and cultured for 24 h before siRNA and drug treatment (see above). Cells were then fixed with 3.7% paraformaldehyde (PFA) for 15 min, and permeabilized with 0.5% Triton X-100 in PBS for 10 min. The cells were then blocked with 3% BSA for 1 h, prior to incubation at 4°C overnight with primary antibodies, including γH_2_AX (Cat. 07-164, Millipore: dilution 1: 400) and 53BP1 (Cat. Sc-22760, Santa Cruz, Dallas, TX; dilution 1:400). On the following day, the cells were washed with PBS and incubated for 1 h with the secondary antibody, including FITC-conjugated anti-mouse antibody (Cat. 715-095-150, dilution 1:400, Jackson ImmunoResearch Labs, West Grove, PA) and TRITC-conjugated anti-rabbit antibody (Cat. 711-025-152, Jackson ImmunoResearch Labs; dilution 1:400). Finally, the cells were mounted with cover slips using mounting media containing DAPI stain before visualization.

### Cell Cycle Analysis

We performed cell cycle analyses by flow cytometry as previously described (Shimada et al., 2003). Cells were briefly harvested after siRNA and drug treatment, washed with PBS, and then fixed in cold 70% ethanol overnight. Further, these cells were stained with 20 ml/mL of propidium iodide and treated with 1 mg/mL of RNase for at least 30 min. The cell cycle distribution was then analyzed using FlowJo 10.5.3.

### Data Acquisition

TCGA provisional data sets were pulled from cBioPortal^1^ between the months of November 2017 and January 2018. The pulled data sets consisted of pancreatic adenocarcinoma (PADC; *n* = 147). Since ALKBH6 is the primary source of interest for this analysis, only individuals with valid RNA Seq V2 RSEM data for ALKBH6 were included. To group individuals as having either low or high expression of *NEIL3*, the *z* scores of each individual were considered. Individuals with a *z* score of ≤-0.5 were placed into the low expressing group while individuals with a *z* score of ≥ 0.5 were placed into the high expressing group. Individuals falling between -0.5 and 0.5 were excluded from analyses. In addition, individuals in the low or high expressing group reporting a null value for OS months were excluded from the group analyses.

### Statistical Analysis

Three independent experiments were performed using immunofluorescence, a comet assay, cell proliferation, and a cell survival assay. Data were statistically analyzed using Student’s *t*-test. Survival analyses of high and low expression of ALKBH6 were evaluated using the Kaplan–Meier method and the log-rank test using a Graph Pad prism. Results were considered significant at *P* < 0.05.

## Results

### Human ALKBH6 Genes Complement ALKB Deficiency in *E. coli* and Enhance Response for DNA Alkylating Agents

**FIGURE 1:**
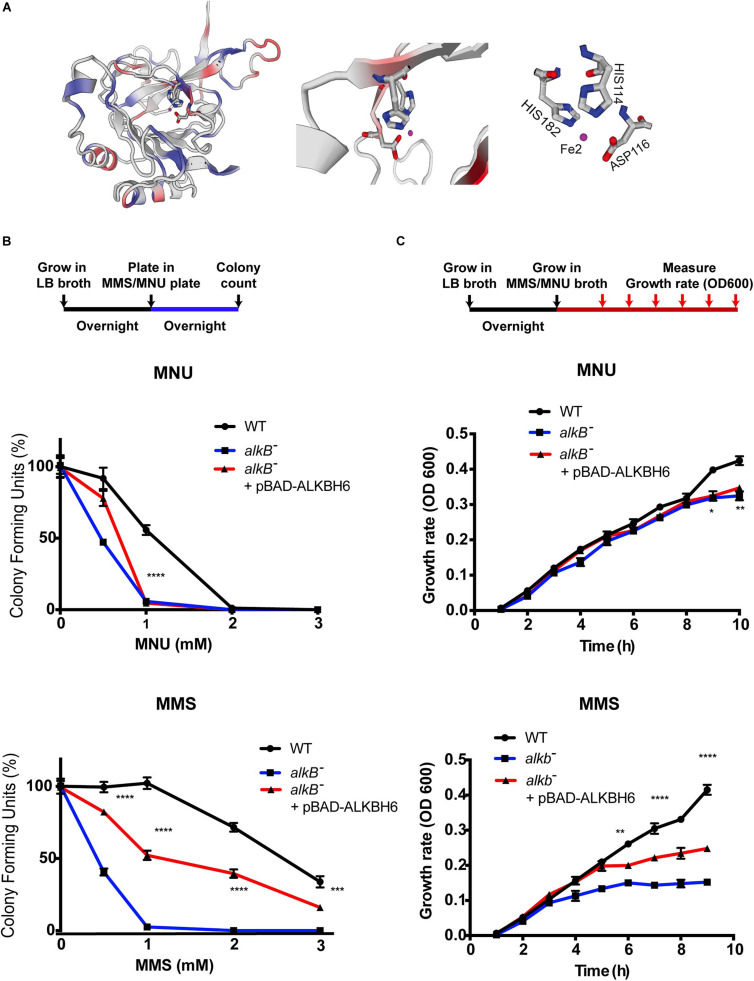
ALKBH6 partially restores growth and increases resistance to alkylating DNA-damaging agents. **(A)** Model of ALKBH6 (238 aa) based on SWISS-MODEL indicating metal binding sites labeled in red (HIS114, ASP116, and HIS182). The Fe^2+^ ion in the active site is shown in purple; protein residues (His114 represented by blue/gray ring; His182 represented by blue/gray ring; D116 represented by gray/red) are labeled in white. **(B)** Schematic representation and results of colony-forming assay using *ALKB*-proficient strain (WT), ALKB-deficient strain (*ALKB*^–^), and ALKBH6-complemented *E. coli* strain (ALKB*^–^* + pBAD-ALKBH6). Results were normalized with untreated groups. Student’s *t*-test was used to determine whether there was significant difference between deficient and complemented strains. **(C)** Schematic representation and results of growth curve assay using the same three strains mentioned above. *p < 0.05, **p < 0.01, ***p < 0.001, ****p < 0.0001 represent *t*-test results. N.S represents non-significant difference, three independent experiments.

Biochemical and structural studies of *E. coli* ALKB and other human ALKBH proteins provide mechanistic insight into how oxidative dealkylation catalyzed by ALKB dioxygenases proteins with an Fe^2+^ binding site at H131, D133, and H187 active sites (Mishina et al., 2004). In addition, the minor β-sheet, and the major β-sheet, contain the 2OG-binding R_20__4_xNxTxR_210_ motif (numbering of the residues as in the ALKB structure). The 1-carboxylate and 2-oxo groups of 2OG bind to the metal in a bidentate manner, whereas the 5-carboxylate of 2OG interacts by means of a hydrogen bond with Arg 210 and by means of a salt bridge with Arg 204 (Yu et al., 2006; Yang et al., 2008; Yi et al., 2010; Feng et al., 2014). However, little is known about the functional domain of ALKBH6 proteins in mammalian cells. Specifically, ALKBH6 contains an Fe (II) metal center in the wild-type enzymes coordinated to the binding site at H114, D116, and H182, with two α-KG binding sites (103–105 aa and 218–22 4aa), and oxygen (Figure 1A, taxonomic identifier 9606, NCBI). To determine whether ALKBH6 plays role in alkylating DNA damage repair, we cloned human ALKBH6 genes and generated an *E. coli* strain to express human ALKBH6 using pBAD-24 plasmids. Our growth assay shows that expression of ALKBH6 in an ALKB-deficient strain increases survival by 50% during 1-mM MMS treatment (Figure 1B). In contrast, ALKBH6 complements ALKB-deficient *E. coli* and enhances survival by 1% during 1-mM MNU treatment (Figure 1C).

### ALKBH6-Deficient Pancreatic Cells Accumulate Alkylating Agent-Induced DNA Damage

**FIGURE 2:**
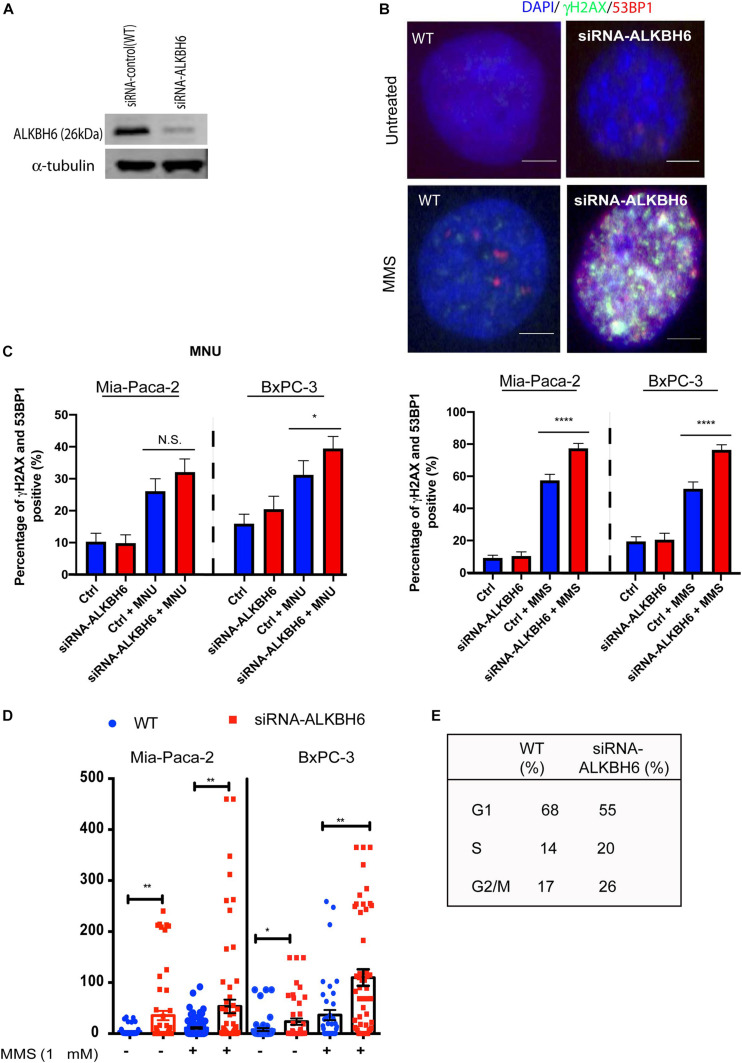
Loss of ALKBH6 induces DSBs. **(A)** Western blot of siRNA-mediated silencing of ALKBH6 on MIA-PaCa-2 cells; **(B)** Representative image of siRNA-mediated silencing of BXPC3 and MIA-PaCa-2 cells treated with MMS and examined with co-localization of 53BP1 and γH2AX; note that blue color represents DAPI staining of the cell nuclei; scale bar represents 10 μm; **(C)** Percent of cells positive for co-localization of 53BP1 and γH2AX treated with MMS/MNU; **(D)** Representative image of ALKBH6 knockout; **(D)** results of tail moment of BxPC3 and MIA-PaCa-2 cells treated with MMS and examined with neutral comet assay; **(E)** Cell cycle profile of cells deficient in ALKBH6. Student’s *t*-test was applied for data analysis. *p < 0.05, **p < 0.01), ****p < 0.0001, N.S represents non-significant difference, three independent experiments.

To determine whether ALKBH6 protects pancreatic cancer cells from accumulating alkylating agent-induced DNA damage, we performed siRNA-mediated silencing of ALKBH6 in BXPC3 and MIA-PaCa-2 pancreatic cancer cell lines and performed an immunofluorescence co-localization assay. Silencing of ALKBH6 with siRNA was confirmed with Western blot which showed a >90% depletion of the target protein (Figure 2A). To further explore whether ALKBH6 protects PDAC cells from alkylating DNA damage, we treated BxPC3 and MIA-PaCa-2 cells with MMS or MNU for 1 h and then examined them by immunofluorescence staining for γH2AX and 53BP1 co-localization (Figure 2B), both established biomarkers for DSBs. We found that siRNA mediated silencing of ALKBH6 in BXPC3 and MIA-PaCa-2 cell lines led to a significantly increased co-localization of γH2AX/53BP1 in the BXPC3 (80%) and MIA-PaCa-2 cells treated with MMS (75%) versus WT cells (40%) (Figure 2C; left panel; *P* < 0.001). In contrast, there was no significant difference found in the cells treated with MNU versus untreated cells (Figure 2C; right panel). Furthermore, the neutral comet assay confirmed that DSBs were significantly increased in siRNA-mediated ALKBH6 silencing in BXPC3 and MIA-PaCa-2 cells following their treatment with MMS (^∗∗^*P* < 0.01) as compared to untreated cells and/or wild-type cells treated with MMS (Figure 2D). Our data suggested that ALKBH6 prevents accumulation of MMS-induced DSBs in PDAC cells.

### ALKBH6 Required for Cell Survival During Exposure to Alkylating Agents

**FIGURE 3:**
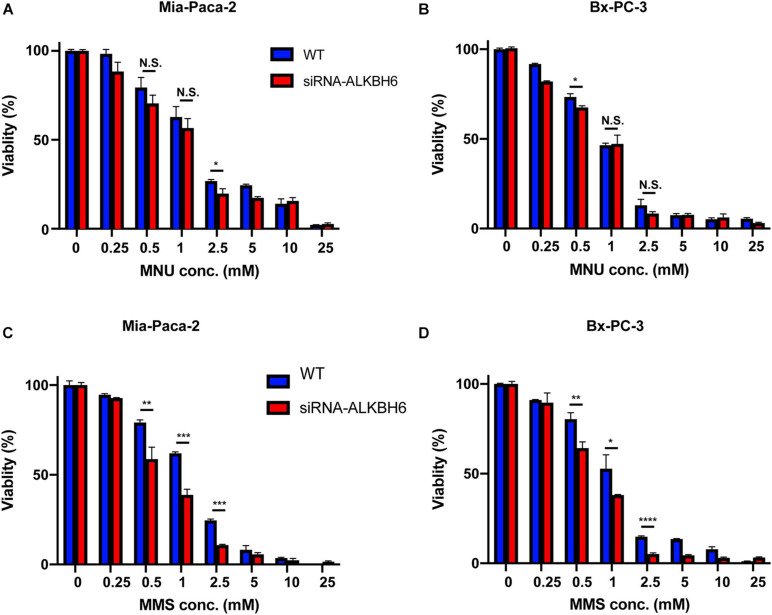
ALKBH6 protects pancreatic cells from alkylating agents. **(A,B)** MTT assay results of MIA-PaCa-2 and BxPC-3 cells treated with a different concentration of MNU for 24 h following ALKBH6 knockout; **(C,D)** MTT assay results of MIA-PaCa-2 and BxPC-3 cells treated with a different concentration of MMS for 24 h following siRNA silencing of ALKBH6 cells; Student’s *t*-test was applied for data analysis. *p < 0.05, **p < 0.01, ***p < 0.001), ****p < 0.0001, N.S represents non-significant difference, three independent experiments.

To determine whether ALKBH6 promotes cell survival and cellular growth, we performed siRNA-mediated silencing of ALKBH6 in BXPC3 and MIA-PaCa-2 pancreatic cancer cell lines and performed a clonogenic survival assay. We investigated whether siRNA-mediated silencing of ALKBH6 in PDAC cells had decreased cell proliferation and increased cell survival after treatment with MMS or MNU (Figures 3A,B). We show that by directly measuring both cell growth and cell survival, we can likely provide evidence on how the pancreatic cells respond to MMS and MNU treatments. Cell viability in the ALKBH6-deficient BXPC3 and MIA-PaCa-2 cells were found to be significantly reduced as compared to the ALKBH6-proficient BXPC3 (60% versus 75%; *P* < 0.01) and MIA-PaCa-2 (55% versus 80%; *P* < 0.01) cells following treatment with MMS, but not with MNU (Figures 3C,D). Collectively, these data indicate that ALKBH6 deficiency leaves PDAC cells vulnerable to cytotoxicity resulting from SN2 alkylating agent treatment.

### ALKBH6 Overexpression in p53 Mutant Tumors Induces Poor Overall Survival in Pancreatic Cancer Patients

**FIGURE 4:**
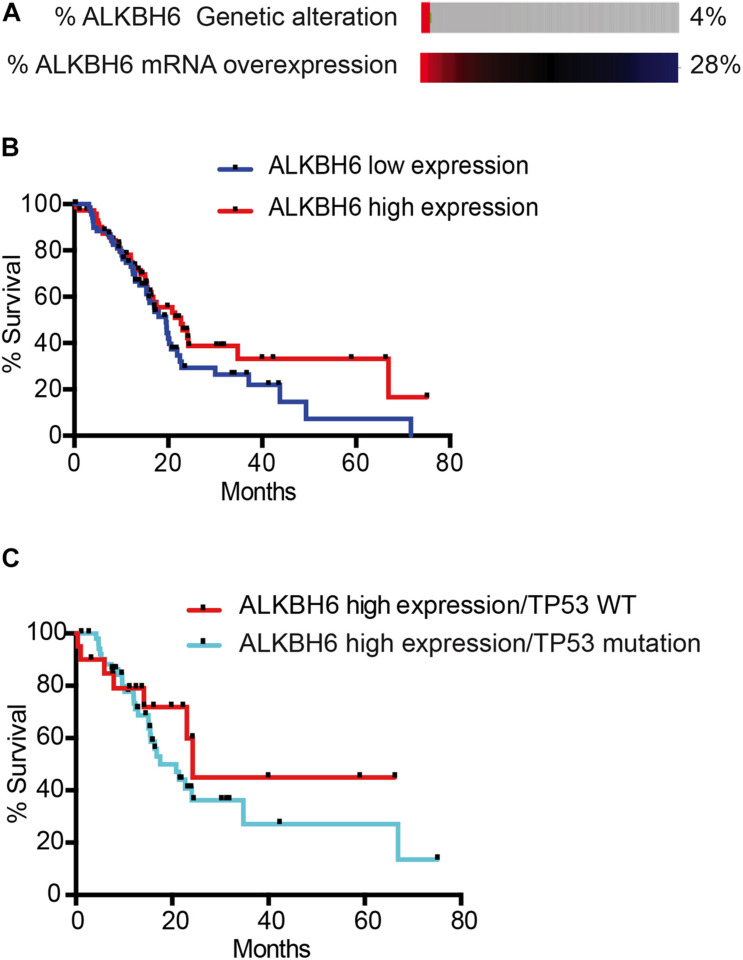
ALKBH6 overexpressed in pancreatic cancer patients positively correlates with patient survival. **(A)** Schematic representation of ALKBH6 gene amplification and expression levels of ALKBH6 mRNA in normal versus tumor samples. mRNA expression was analyzed from TCGA database and the RNA Seq dataset was analyzed using the log scale expression. An expression of >0.5 denotes the high expressed group (*n* = 76) versus < –0.5 for the low expressed group (*n* = 71); **(B)** Survival curve of pancreatic cancer patients with high ALKBH6-expressing versus low ALKBH6-expressing pancreatic tumors. Kaplan-Meier survival graph of patients with high and low expression of ALKBH6; **(C)** Survival of patients with (*n* = 56) and without (*n* = 20) TP53 mutation-driven ALKBH6-overexpressing tumors promotes worse prognosis in pancreatic cancer.

To correlate our findings to human pancreatic cancer, we quantified the expression data of ALKBH6 in pancreatic cancer using The Cancer Genome Atlas database (TCGA). We observed that 28% of pancreatic cancer patients overexpressed mRNA of ALKBH6 in tumor tissue samples relative to normal pancreatic tissue (Figure 4A). Moreover, high expression of ALKBH6 in pancreatic cancer tissue was correlated with favorable overall patient survival (Figure 4B). Similar favorable overall survival outcomes were observed in head and neck cancer and skin cutaneous carcinoma. In contrast, we found that the majority of tumor tissues that significantly overexpressed ALKBH6 were associated with poor overall survival in a few types of cancer including low-grade glioma (LGG) and kidney renal clear cell carcinoma (data not shown). Furthermore, mutation in p53 in ALKBH6-overexpressing pancreatic cancer patients significantly decreased the rate of overall survival (Figure 4C). Our results show that co-occurrence of ALKBH6 overexpression and loss of tumor suppressor genes status is critical for pancreatic cancer progression and negatively impacts patient overall survival.

## Discussion

Environmental risk factors and metabolic stress within the cell likely induce alkyl and methyl DNA and/or RNA damage. The repair of DNA or RNA base lesions is critical to maintain normal cellular functions, including genomic stability. It is important to identify the biological significance of the mammalian proteins involved in the repair of DNA alkylation adducts. Alkylating agents induce different types of DNA base lesions, and each substrate is likely repaired by different types of enzymes to prevent mutagenesis (Sedgwick et al., 2007; Fu et al., 2012). Humans have nine different ALKBH proteins, with some involved in direct repair of alkylating DNA damage. However, the type of substrates used for the transfer of alkyl groups determines which ALKBH protein is involved at the DNA base lesions (Li et al., 2013), thus indicating the importance of different types of oxidative demethylation for cell survival. In this study, we have demonstrated that ALKBH6 partially complements ALKB-deficient *E. coli* and restores survival and growth, following the SN2 type of alkylating DNA damage-induced cytotoxicity. Similarly, previous studies have demonstrated that ALKBH2 and ALKBH3 contribute to the repair of alkylating DNA damage and restore cellular survival of *E. coli* ALKB-deficient cells *in vitro* (Falnes et al., 2002; Trewick et al., 2002; Aas et al., 2003; Tasaki et al., 2011). Further, ALKBH2 and 3 complement ALKB-deficient E. coli strains and restore the transformation efficiency of plasmids bearing MMS-induced DNA damage up to 90% (Mishina et al., 2004). In particular, our data show that the loss of ALKBH6 increased MMS-induced DSBs and enhanced the cytotoxicity of MMS in human pancreatic cancer cells. In contrast, no significant difference was observed in pancreatic cells treated with SN1 type alkylating agents (MNU). It is also possible that DSBs in ALKBH6-deficient pancreatic cells likely arise from unrepaired SSBs that carry over to the S-phase of the cell cycle. Alternatively, it is also possible that a loss of ALKBH6 causes the accumulation of SSBs in close proximity to replication forks or processing of the DNA end-blocking groups with nucleases (Ma et al., 2009), which likely contributes to MMS-induced DSBs. Similarly, our data support that MMS-induced DNA base lesions in ssDNA are repaired by EcALKB *in vivo* and *in vitro* (Dinglay et al., 2000). Further, previous works have shown that ALKBH1-3 protect cells against MMS-induced damage (Ougland et al., 2004; Vagbo et al., 2013) and repair m^1^A and m^3^C DNA base lesions, preventing replication and transcription-related stress that could trigger cell cycle checkpoint activation and apoptosis (Wei et al., 1996; Duncan et al., 2002; Aas et al., 2003; Falnes et al., 2004; Westbye et al., 2008). It is known that both chemicals actually cause a similar range of methylated residues. The only substantial difference in dsDNA is that MNU methylates much more backbone phosphate and O6 guanine residues, compared to MMS, with the latter causing slightly increased 1 meA and 3 meA levels. On the other hand, in ssDNA, 1 meA and 3 meC appear to much more apparent in MMS-treated cells. However, our observations are in contrast with other DNA damaging agents, such as ionization radiation (Ward, 1994) which causes clustered DNA damage in close proximity. Consistent with the above evidence, results of this study suggest that ALKBH6 is critical in protecting pancreatic cancer cells from MMS-induced DNA base damage. Therefore, treatment of cells with MMS may lead to an increase in substrates recognized by ALKBH6. Further studies are warranted to define the precise role of ALKBH6 to protect mutagenesis-mediated genomic instability.

Our data demonstrated that there is no significant difference in spontaneous DNA damage between siRNA-silenced ALKBH6 cells and wild-type pancreatic cancer cells. However, siRNA-silenced ALKBH6 leads to the accumulation of cells in the G2/M phase of the cell cycle (Figure 2E) suggesting that ALKBH6 may contribute to the mitotic cellular growth of pancreatic cancer cells. Further, our data show that siRNA-mediated silencing of ALKBH6 impacts cell viability. In contrast, silencing of ALKBH2, 3, and 8 or both ALKBH2 and 3, did not affect cancer cell viability (Pilžys et al., 2019). Our results also demonstrated that MMS treatment compromises the survival of pancreatic cancer cells, suggesting the involvement of ALKBH6 in DNA repair. These findings are in agreement with previous studies, showing that deletion of ALKBH repair results in reduced cell survival (Ringvoll et al., 2006; Sikora et al., 2010; Dango et al., 2011; Pilžys et al., 2019).

Considering the clinical relevance of our study, ALKBH6 is significantly overexpressed in pancreatic cancer and is associated with favorable overall survival. In addition, a high level of ALKBH6 expression has been detected in adenocarcinomas of other organs, such as head and neck squamous cell carcinoma, malignant melanoma, and renal cell carcinoma, with favorable overall survival as well. In contrast, overexpression of ALKBH6 is associated with poor overall patient survival in LGG. The relatively high expression of ALKBH6 in tumor tissues correlate with cancer development in some cancer types, and may provide an opportunity to use ALKBH6 as a potential target for anticancer therapy (Chen et al., 2019; Li et al., 2019). Other members of the ALKBH family, such as ALKBH3, are essential for cancer progression and present themselves as potential targets for effective therapy in prostate cancer (Konishi et al., 2005; Shimada et al., 2008). In addition, ALKBH2 plays a crucial role in the treatment of pediatric brain tumors during chemotherapy (Cetica et al., 2009). In summary, our results suggest that ALKBH6 is involved in protecting pancreatic cancer from alkylating-induced DNA damage and promotes cell survival. Further biochemical characterizations are required to determine whether ALKBH6 repairs m^1^A or m^3^C. Our data provide a basis for future studies to uncover the clinical benefit of ALKBH6 in certain cancers as a prognostic marker or a potential target for therapy.

## Data Availability Statement

The original contributions presented in the study are included in the article/supplementary material, further inquiries can be directed to the corresponding author/s.

## Author Contributions

SZ perform the experiment. RD perform DNA damage experiment on *E. coli.* AF perform DNA damage localization in human pancreatic cancer cells. DK and SZ perform data analysis and wrote the manuscript. All authors contributed to the article and approved the submitted version.

## Conflict of Interest

The authors declare that the research was conducted in the absence of any commercial or financial relationships that could be construed as a potential conflict of interest.

## Footnotes

1 http://cbioportal.org
